# Maternal Separation during Breastfeeding Induces Gender-Dependent Changes in Anxiety and the GABA-A Receptor Alpha-Subunit in Adult Wistar Rats

**DOI:** 10.1371/journal.pone.0068010

**Published:** 2013-06-27

**Authors:** Diego Armando León Rodríguez, Zulma Dueñas

**Affiliations:** 1 Facultad de Medicina, Universidad Nacional de Colombia, Bogotá, Colombia; 2 Departamento de Ciencias Fisiológicas, Facultad de Medicina, Universidad Nacional de Colombia, Bogotá, Colombia; Radboud University, The Netherlands

## Abstract

Different models of rodent maternal separation (MS) have been used to investigate long-term neurobiological and behavioral changes, associated with early stress. However, few studies have involved the analysis of sex-related differences in central anxiety modulation. This study investigated whether MS during breastfeeding affected adult males and females in terms of anxiety and brain GABA-A receptor-alpha-subunit immunoreactivity. The brain areas analyzed were the amygdale (AM), hippocampus (HP), medial prefrontal cortex (mPFC), medial preoptic area (POA) and paraventricular nucleus (PVN). Rats were housed under a reversed light/dark cycle (lights off at 7∶00 h) with access to water and food *ad libitum.* Animals underwent MS twice daily during the dark cycle from postnatal day 1 to postnatal day 21. Behavior was tested when rats were 65–70 days old using the elevated plus maze and after brains were treated for immunohistochemistry. We found that separated females spent more time in the open arms and showed more head dipping behavior compared with controls. The separated males spent more time in the center of the maze and engaged in more stretching behavior than the controls. Immunohistochemistry showed that separated females had less immunostained cells in the HP, mPFC, PVN and POA, while separated males had fewer immunolabeled cells in the PFC, PVN and AM. These results could indicate that MS has gender-specific effects on anxiety behaviors and that these effects are likely related to developmental alterations involving GABA-A neurotransmission.

## Introduction

Early adverse experiences have been proposed as having long lasting deleterious effects on shaping brain networks and behavioral development in mammals [1]–[4]. For example, children who have experienced prolonged maltreatment, abuse or neglect rearing, use to have an increased risk of developing psychopathology like anxiety disorders, depression, attention deficit [5], learning problems and so on. They are more likely to show heightened neuroendocrine responsiveness to stress, brain morphology changes, and neurochemical and gene expression patterns in the central nervous system related to the emergence of several psychopathologies [6], [7], [8]. In this sense, Maternal Separation (MS) is one of the paradigms to experimentally study the endocrine, behavioral and brain structural consequences of early life stress in animal models [9], [3]. Mammals need care of their mothers to supply them with warmth, food, sensorial stimulation, endocrine regulation, and modulated emotional arousal; separation from mothers, cause high stress and when this stress is recurring during breastfeeding period, may carry to deleterious effects on behavior and neuroendocrine development. Regarding anxiety, the results are not conclusive; some studies have found that animals subjected to MS show increased anxiety-like behavior and related neuroendocrine alterations [10]–[12]; while other researchers have not reported such alterations [13] or have shown a reduction in anxiety in females but not in males [14], [15].

Anxiety responses are mediated by the hypothalamic-pituitary-adrenal axis (HPA), whose activity is modulated by limbic structures like hippocampus, prefrontal cortex, amygdala and parahypothalamic areas. Neurobiological activity begins with corticotropin-releasing hormone (CRH) secretion from the cells of the hypothalamic parvocellular paraventricular nucleus (pPVN), pPVN cells receive GABAergic inhibitor input from limbic structures, such as the medial preoptic area (mPOA), the bed nucleus of the *stria terminalis* (BNST), the dorsomedial hypothalamus (DMH) and the nucleus of the solitary tract (NST) [16]. In turn, related structures before, are regulated by the amygdala, hippocampus and medial prefrontal cortex (mPFC), which have extensive glucocorticoid receptors (GR) forming a stress control circuitry. In this regard, pPVN cell activity is modulated by increasing or reducing the inhibitory input from limbic areas in which GABAergic synapses are especially important, particularly those mediated by GABA-A receptors.

**Figure 1:**
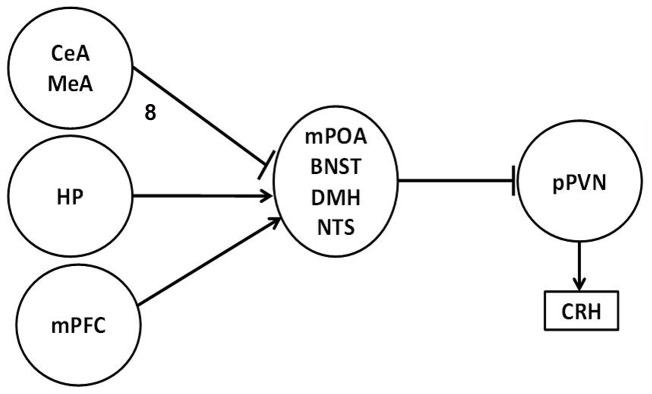
Sketch of PVN limbic brain regulation. The arrows show glutamatergic excitatory signals, and blunt end lines indicate GABAergic inhibitory synapses. Medial amygdaline nucleus (MeA), central amygdaline nucleus (MeA), hippocampus (HP), medial prefrontal cortex (mPFC), medial preoptic area, the bed nucleus of the *stria terminalis* (BNST), dorsomedial hypothalamus (DMH), the nucleus of the solitary tract (NST), parvocellular paraventricular nucleus and corticotropin release hormone (CRH); adapted from Jankord and Herman [17].

Hippocampus and mPFC play a inhibitory role on HPA activity; prelimbic and cingulated mPFC send excitatory projections (glutamatergic) to peri-PVN areas and the BNST, both of which send direct inhibitory (GABAergic) projections to the pPVN: while the hippocampus sends excitatory projections to the posterior BNST, peri-PVN regions, mPOA and DMH, all of which send GABAergic projections to the PVN. On the other hand, amygdala increases de HPA activity: the medial and central amygdaline nuclei (CeA and MeA) send inhibitory projections to GABAergic PVN-projecting populations, such as the BNST, mPOA and peri-PVN, eliciting trans-synaptic disinhibition [17]. Figure 1 illustrates this excitatory and inhibitory interaction.

Repeated early MS has been reported to produce structural disruption in above limbic areas. Structural and physiological disruption in hippocampus has been found in rodents, especially in CA1 and CA3 neuron density and synapsis formation [18], [19], [20], [21], [22]. In relation with mPFC, mice and rats separated from their mothers present changes in neuron and glia development [23], [24], which may carry cortical hypofunction associated whit GABA transmission disruption [25], [26]. Concerning the amygdala, GABA-A and benzodiazepine (BZ) binding site alterations have been reported [10]. Few studies have showed the consequences of early stress on the intermediate nucleus; Sánchez *et al*. [27] have shown that neonatally isolated rats had reduced neuronal activity in the pPVN, mPOA and BNST; however, it should be noted that the effects of early stress on these areas could have specific gender-related effects [28], [29].

Some studies have explored the specifics gender-related effects of MS, for example; Farkas and coworkers [30], found that 3 hour of daily separation produced gender specific changes in motor development, Slotten et al. [31] found that MS cause long-term gender dependent effects on motor activity, open field exploration and basal plasma corticosterone. Eklund and Arborelius [14] showed that early maternal separation produced a reduction in anxiety-like behaviors in females but not in males, and León and Dueñas [15] reported that separated females, but not males, show less anxiety behavior in elevated plus maze.

In summary, MS have effects on neurobehavioral development, in particular on anxiety behaviors, which may be sex specific. But still is unclear if there is any relation between early MS, GABA-A neurotransmission in limbic loop that regulates pPVN activity, and anxiety-like behaviors. Thus, the goal of this study was to investigate the consequences of early MS on anxiety-like behavior and GABA-A receptor immunoreactivity in the mPFC, hippocampus, amygdala, mPOA and pPVN in adults males and females.

## Materials and Methods

### 1. Animals

Female and male Wistar rats were obtained from the Universidad Nacional de Colombia’s in-house animal facility at its veterinary school. Animals were housed in standard rat cages (40 cm×31 cm×22 cm) and maintained under standard laboratory conditions with a reversed light/dark cycle (lights off at 07∶00 and on at 19∶00 hours), room temperature of 22±2°C, 55±10% humidity and food and water provided *ad libitum.* Six dams were allowed to mate separately with one male and then each dam was single-housed until the parturition. Each litter consisted of 10 subjects that were randomly assigned to either the separation or animal-facility-reared (AFR) groups, attempts were made to try to achieve a balanced distribution for gender. All experimental protocols were approved by the Universidad Nacional de Colombia’s Ethics Committee and conformed to the National Institutes of Health Guide for the Care and Use of Laboratory Animals.

### 2. Maternal Separation Procedure

The litters were undisturbed at birth (P0). The mothers were first removed from their home-cage in the main colony room and placed into a fresh cage at P1. The pups were subsequently sexed and randomly assigned to undergo MS or animal-facility-reared (AFR) procedures. In each separation pups of the same litter were placed together in a small cage being separated from their dam and ARF siblings and then removed to other room to prevent communication by means of odors or ultrasonic vocalization with their mothers [32]. The separated pups were placed on a heating pad set at 25–28°C for 6 h (3 h in the morning and 3 h in the afternoon) from P1 to P21. The pups were then returned to the original home-cage in the main colony room after the 3 h separation, immediately followed by the mother’s return. The no separated siblings remained undisturbed except for routine weighing each two days and cage cleaning performed once a week, for these reason are no considered control group but AFR. The separation procedure was carried out at the same time every day (7∶00–10∶00 and 13∶00–16∶00). The litters were weighed every two days until P21. Males and females were weaned and group-housed according to gender and treatment at P22 (7–8 per cage). The rats remained undisturbed until adulthood and were never alone in the cage to prevent deleterious effects of stress due to social deprivation. All subsequent experiments were performed during adulthood (65–70 days old).

### 3. Elevated Plus Maze Test

The elevated plus maze (EPM) is a useful tool for testing anxiety. This instrument allows for unconditioned fear/anxiety-like tendencies to be measured and is also sensitive to locomotor activity and decision-making patterns [33], [34]. The EPM was made from dark black acrylic material and consisted of two open arms (50 cm×10 cm) crossed at right angles with two opposed arms of the same size. Two of the opposed arms were enclosed by 40-cm high walls except for the central portion where the arms crossed. The whole apparatus was raised 50 cm above the floor. A plexiglas rim (1 cm high) surrounded the perimeter of the open arms to prevent the rats from falling off.

To behavioral measurements were used forty-eight subjects distributed in following groups: 10 AFR females, 11 separated females, 16 AFR males and 11 separated males. Experimental sessions occurred during the dark phase in a dark room and were recorded using an infrared video camera interfaced with a monitor and a DVD in an adjacent room. A rat was placed into the central area of the maze facing one of the open arms at the beginning of each session and allowed free-exploration for 5 min. The female rats were tested to determine their estrus cycle by examining vaginal smear; the test was carried in the morning and the behavioral measurement in the afternoon allowing the females rest. All female animals were evaluated during their diestrus phase. The results are expressed as time spent in the open arms, total entries and time in the center. An entry was scored when all four paws entered a single arm. The frequency and time spent on the following behaviors were also measured: head dipping (sticking the head outside the maze border and below floor level, negatively correlated with anxiety) and stretching (elongating the body whilst keeping the hind paws fixed; closed arm stretching is positively correlated with anxiety, while open arm stretching correlates with approach-avoidance conflict). The behavioral test was always carried out between 14∶00–16∶00 h and was applied to all 48 pups according to the distribution mentioned above. Free specially designed software for recording behavior (X-PloRat) was used for behavioral analysis.

### 4. Perfusion and Tissue Processing

These procedures were performed one day after the EPM test. The rats were anaesthetized with chloral hydrate (45 mg/100 g body weight) and intracardially perfused with saline (0.9%) followed by 4% paraformaldehyde in 0.1 M PBS. The brains were dissected out and post-fixed with the same fixative solution for 48 h at 4°C. Following fixation, the brains were cryoprotected in a 30% sucrose solution for at least 48 h and then covered with tissue freezing medium. Twenty four brains were processed for immunohistochemistry (IHC) (6 MS and AFR males and 6 MS and AFR females, one from each dam) and the remaining brains were stored. Coronal sections (20-µm thick) were cut on a cryostat (Leica CM1850). Five series of brain sections from the prelimbic area of the medial prefrontal cortex (mPFC) bregma 2.70 mm, preoptic area (POA) bregma 4.30, paraventricular nucleus (PVN) bregma −0.40, central and medial nucleus of the amygdala (AM) bregma −3.14 and CA3 of the hippocampus (HP) bregma −3.14 were used for immunohistochemistry.

### 5. Immunohistochemistry

Brain sections from both AFR and experimental rats were selected according to anatomical landmarks identified using the Paxinos [35] rat brain atlas that corresponded to the selected areas. The sections were simultaneously processed in a free-floating state for GABA-A α1 subunit detection. The brain sections were treated for 1 h with 3% v/v normal goat serum in phosphate-buffered saline (PBS 0.1 M) containing 1.5% BSA and Triton x-100 to block nonspecific binding sites. Afterwards, the sections were incubated for 12 h with an anti-GABA-A α1 subunit antibody (Sigma G 4416), diluted 1∶250 in PBS-BSA. After five rinses in PBS (15 min each), the sections were incubated for 1 h at room temperature with biotinylated secondary antibodies diluted 1∶500 (Vectastain kit, Vector USA). After further washes in PBS, sections were incubated for 1 h with a streptavidin-peroxidase complex diluted 1∶500. The sections were then washed five times in PBS and twice in 0.9% NaCl buffer. Peroxidase activity was demonstrated using diaminobenzidine hydrochloride (DAB, included in the same kit). Sections were washed three times with saline solution at the end of the enzymatic reaction step. The sections were then mounted on gelatin-coated slides, air-dried and coverslipped using cytoresin for light microscope observation.

### 6. Quantifying Immunolabeled Cells

All images were captured using a light microscope (Carl Zeiss-AxioVert 40 CFL) and a digital camera (Cannon Power Shot 640). The profiles were counted in representative serial sections (a minimum of four sections per brain area per rat). The number of labeled cells from 250 µm^2^ regions per area were counted manually using ImageJ software. The investigators were blind to the grouping while taking the photomicrographs and performing the image analysis. All images used in the analysis were taken on the same microscope and at the same optical settings.

### 7. Statistical Methods

The data were analyzed using Sigma Stat 3.5 for Windows XP, and a two-way ANOVA analysis of variance (treatment×gender) and the Holm-Sidak post-hoc test were used for multiple comparisons. Significant differences were estimated at p<0.05.

## Results

### 1. Litter Weight Dynamics

**Figure 2:**
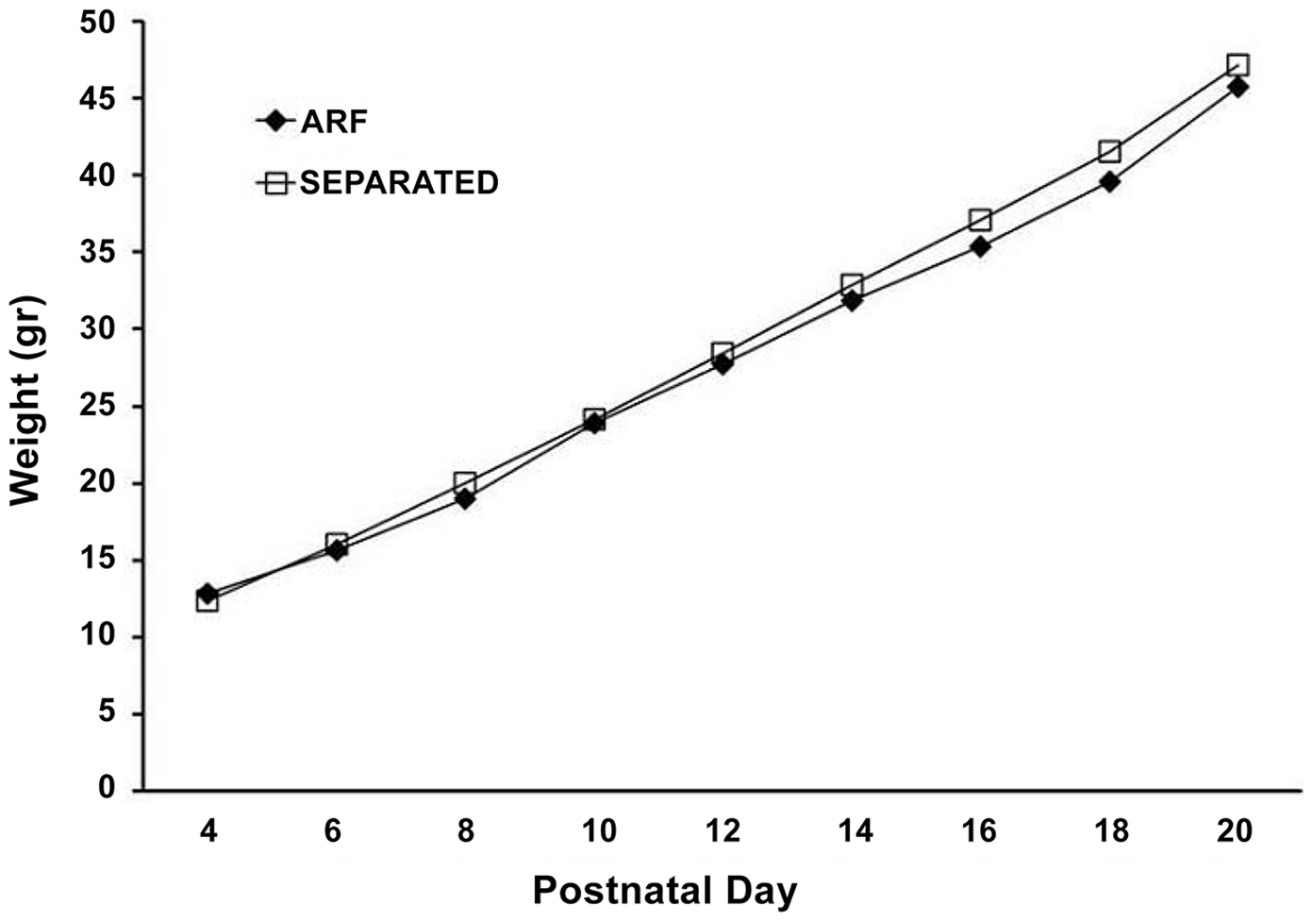
Litters dynamic weights in both maternal separated and non-separated groups. Each point corresponded to the means of ARF and separated subjects: PND 4 (t* = *0.81, p* = *0.422), PND 6 (t* = *0.5711, p* = *0.571), PND 8 (t* = *1.065, p* = *0.292), PND 10 (t* = *0.149, p* = *0.882), PND 12 (t* = *572.5, p* = *0.495), PND 14 (t* = *578.5, p* = *0.42), PND 16 (t* = *1.324, p* = *0.192), PND 18 (t* = *1.128, p* = *0.133), PND 20 (t* = *0.739, p* = *0.464), n = 48.

To verify the normal development of the pups, all animals were weighed every two days from postnatal day (PND) 4 until PND 20. In figure 2, we show the litter weight dynamics from the MS pups and controls. We did not find any differences in the body weights between groups.

### 2. Elevated Plus Maze Test

As shown in Table 1, females made more open arms entries, spent more time in these arms, made more total arm entries and spent more time in dipping behaviors, while males spent more time in maze center and in stretching behaviors. Concerning treatment factor, maternally separated animals had more open arms entries, spent more time in open arms, made more total entries, stayed more time in maze center, made more dipping and stretching behaviors. The test showed significant interaction between gender and treatment factor, the only category in which there was no significant interaction was the total number of arm entries.

**Table 1:** Anovas for Behavior and Immunohistochemistry.

		Gender		Treatment		Interaction	
Measurement		*F*	*P*	*F*	*P*	*F*	*P*
Behavior	% open arm entry	39.84	<0.001*	4.374	0.042*	6.537	0.014*
	Time in open arms	58.979	<0.001*	13.984	<0.001*	15.887	<0.001*
	Total entries	18.479	<0.001*	31.628	<0.001*	2.71	0.107
	Time spent in center	5.368	0.025*	7.285	0.01*	4.92	0.032*
	Time spent dipping	13.805	<0.001*	43.123	<0.001*	5.518	0.023*
	Time spent stretching	53.44	<0.001*	4.389	0.042*	21.56	<0.001*
IHC	Amygdala	0.279	0.603	16.850	<0.001*	0.515	0.481
	Hippocampus	0.541	0.470	32.294	<0.001*	2.507	0.129
	Prefrontal Cortex	2.741	0.113	32.762	<0.001*	0.166	0.688
	Paraventricular nucleus	0.00781	0.930	61.954	<0.001*	9.429	0.006*
	Preoptic area	4.595	0.045	73.677	<0.001*	21.446	<0.001*

Two-way ANOVA results for each behavior (n = 48) and the immunohistochemistry data (n = 24). *statistically significant differences p<0.05.

#### 2.1. Differences between genders into treatment

**Figure 3:**
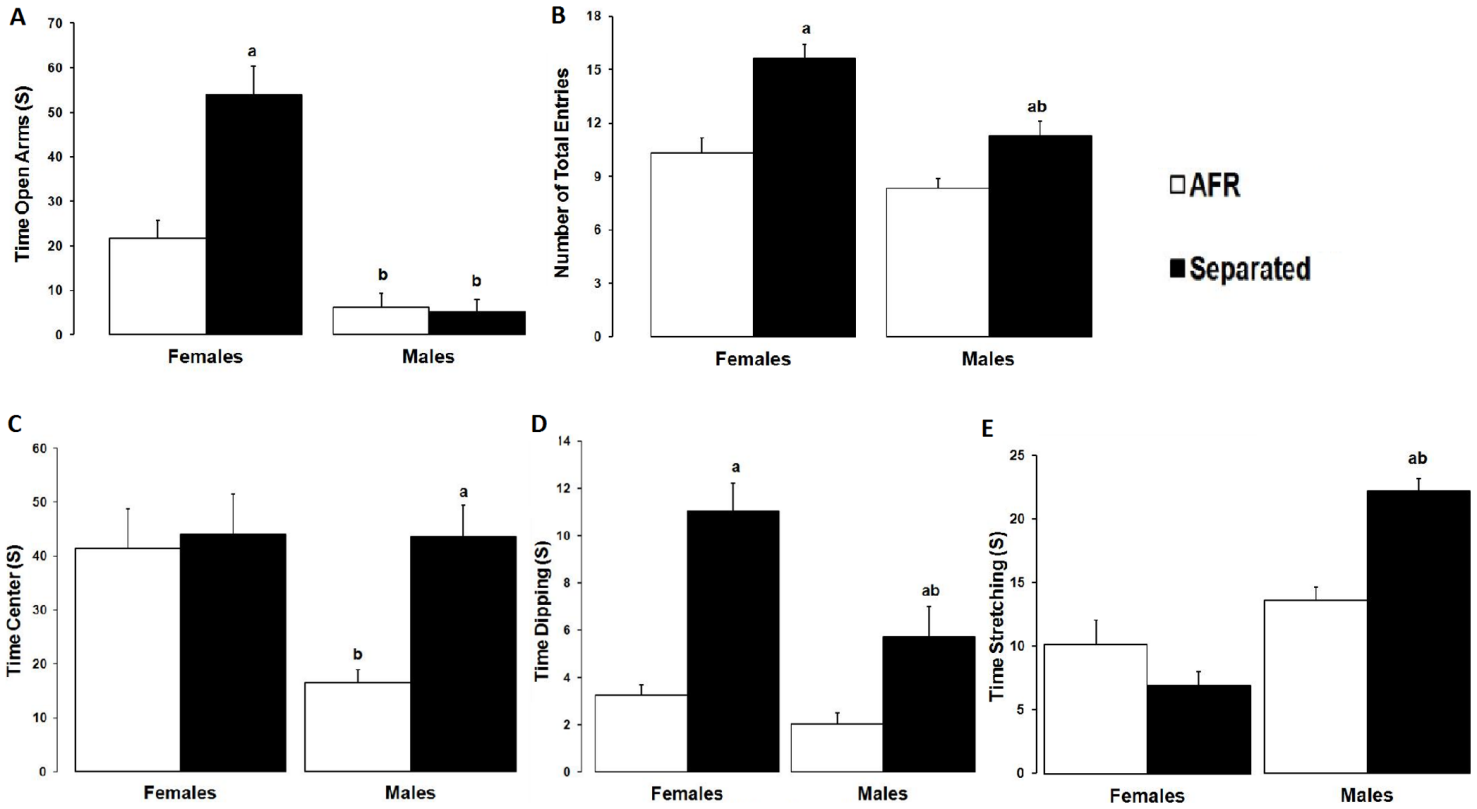
Elevated plus maze results according to gender (females = left, males = right) and treatment (AFR = white, MS = black). (**a**) Time spent in the open arms: AFR vs. MS females (t = 5.160, p* = *0.0001); AFR females vs. AFR males (t* = *2.705, p* = *0.010); MS females vs. MS males (t* = *7.984, p* = *0.001). (**b**) Number of arm entries: MS vs. AFR females (t* = *4.856, p* = *0.001); AFR vs. MS males (t* = *3, p* = *0.004); MS females vs. MS males (t* = *4.069, p* = *0.00). (**c**) Time spent in the maze center: AFR vs. MS males (t* = *3.708, p* = *0.001); AFR females vs. males (t* = *3.321, p* = *0.002). (**d**) Time spent head dipping: AFR vs. MS females (t* = *5.955, p* = *0.00); AFR vs. MS males (t* = *3.181, p* = *0.003); MS females vs. MS males (t* = *4.150, p* = *0.00). (**e**) Time spent stretching: AFR vs. MS males (t* = *5.082, p* = *0.00); MS females vs. MS males (t* = *8.181, p* = *0.00). a: significant difference was observed between same gender; and b: significant difference between same treatment, p<0.05 in Holm-Sidak test, n = 48.

As can be observed in figure 3a ARF and MS females spent more time into open arms that their males counterpart. Concerning number of total entries, there were no differences between ARF (t = 1,942, p = 0,058) but in MS animals (Figure 3b). ARF females stayed for more time in the center maze that ARF males (Figure 3c), while there were no differences into MS group (t = 0,677, p = 0,946). There were no significant differences into ARF animals in dipping (t = 1,001, p = 0,322) and stretching behaviors (t = 1953, p = 0,057) but into MS, MS females had more dipping and males more stretching (Figure 3d and 3e).

#### 2.2. Differences between treatments into gender

*ARF vs MS Females*: Like is showed in figure 3a and 3d, maternal separated females were the group that showed more anxiolytic behaviors: their stayed significantly more time in open arms and spent more time in dipping behaviors. MS females made more total arms entries than ARF females (Figure 3b). There were no main differences between ARF and MS concerning time in maze center (t = 0,321, p = 0,749) and stretching behavior (t = 1,702, p = 0,096). *ARF vs MS Males*: MS males made more number of total entries (Figure 3b), spent more time in maze center (Figure 3c), did more dipping and stretching behavior (Figure 3d and 3e). The only measurement in which there were no significant differences between males was time into open arms (t = 0,186, p = 0,853).

### 3. Immunohistochemistry

Counting immunostained cells against GABA-A receptor alpha 1 subunit showed no differences between males and females (Table 1). Concerning treatment there were main effect; analysis shed a significant reduction of quantity of inmunostained cells in all brain areas of MS animals (Table 1). The interaction between gender and treatment just was essentially in paraventricular nucleus and preoptic area (Table 1).

#### 3.1. Differences between genders into treatment

There were no significant differences between males and females into ARF animal in AM (t* = *0,134, p* = *0,895), mPFC (t* = *0,883, p* = *0,388), HP (t* = *0,599, p* = *0,556) and POA, only were significant differences in PVN (t* = *2,109, p* = *0,048) where females showed more stained cells that males. Into MS animals were not observed significant differences in AM (t* = *0,202, p* = *0,844), mPFC (t* = *1,459, p* = *0,160), and HP (t* = *1,640, p* = *0,117), but in PVN (t* = *2,234, p* = *0,037) and POA (t* = *4,790, p* = *0,000) in these limbic areas females had lesser stained cells that males.

#### 3.2. Differences between treatments into gender

**Figure 4:**
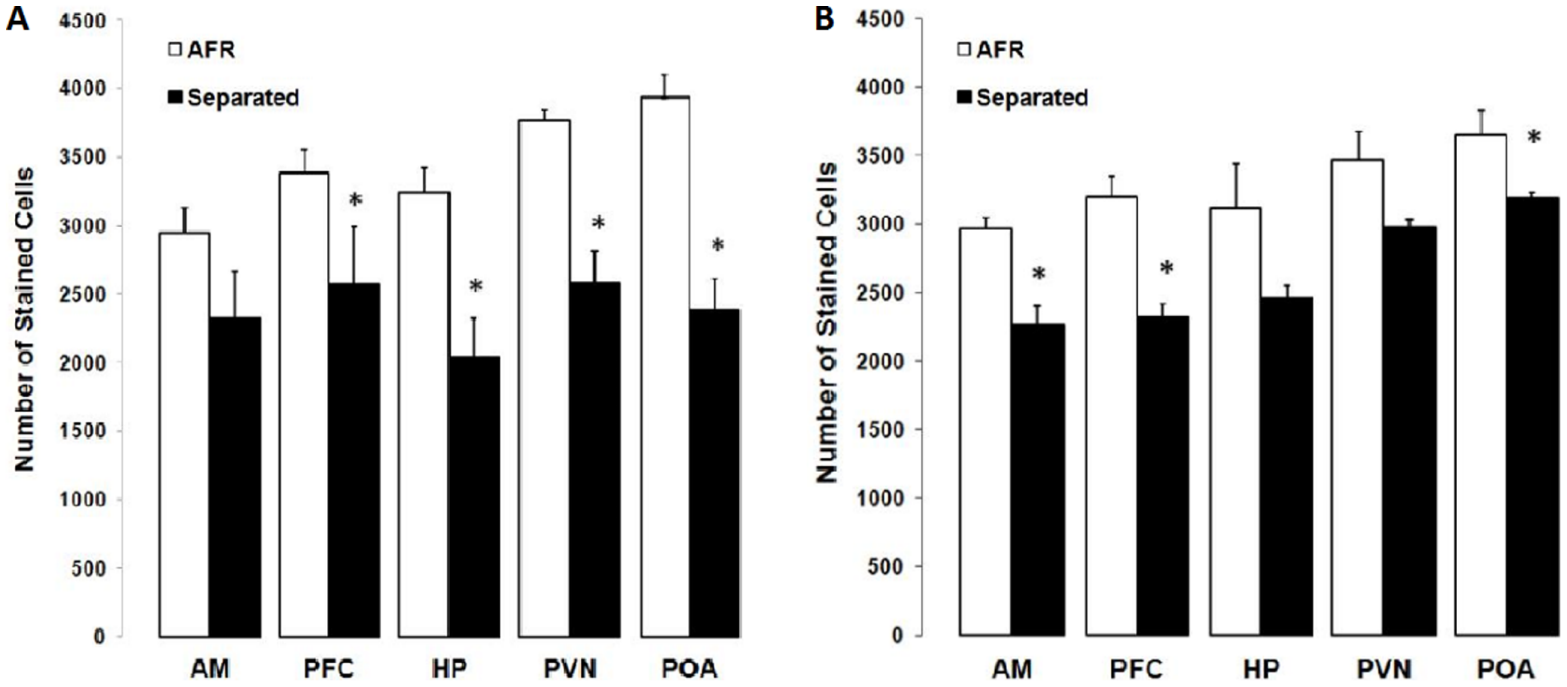
Number of stained cells in the amygdala (AM), prefrontal cortex (PFC), hippocampus (HP), paraventricular nucleus (PVN), preoptic area (POA) according to treatment (AFR = white, MS = black). (a) Females: PFC (t* = *3,759, p* = *0,001), HP (t* = *5,138, p* = *0,000), PVN (t* = *7,737, p* = *0.00) and POA (t* = *7.015, p* = *0.00). (b) Males: AM (t* = *3,410, p* = *0,003), PFC (t* = *4,335, p* = *0,000), PVN (t* = *3,394, p* = *0,003).* significant differences p<0.05 in Holm-Sidak test, n = 24.

**Figure 5:**
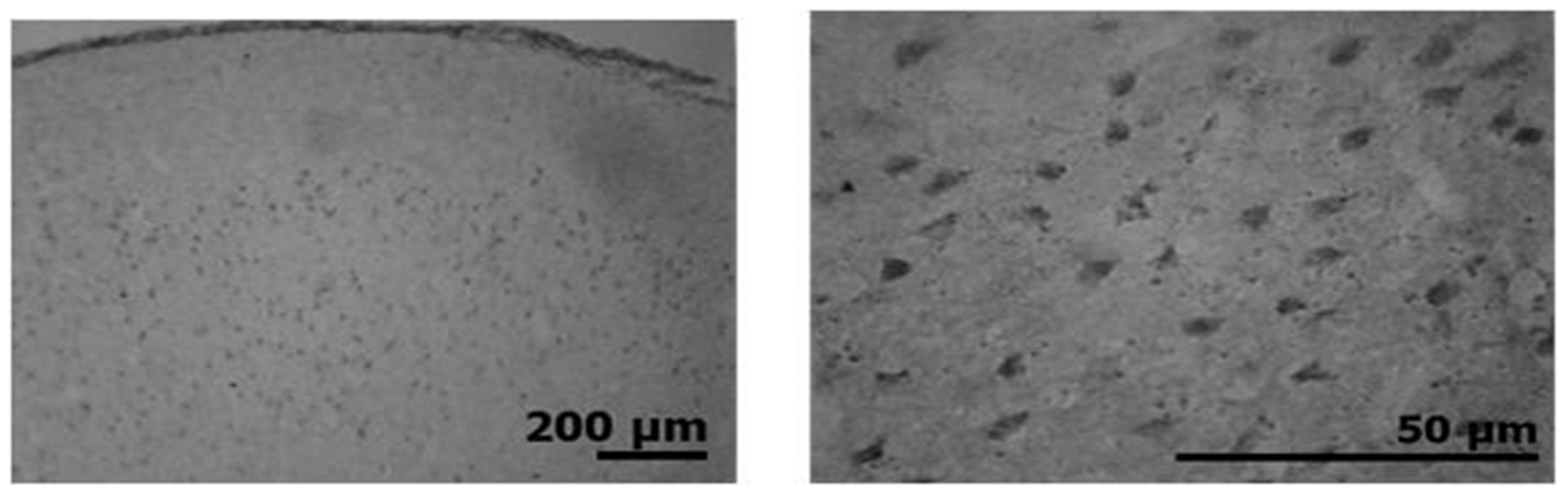
Representative image of immunostaining for the GABA A receptor alpha subunit. The image corresponds to prelimbic area of the medial prefrontal rat cortex (bregma 2.70 mm) immunohistochemistry.

The MS females showed a significant reduction in stained cells in mPFC, hippocampus, pPVN and mPOA compared to AFR females (Figure 4a), while MS males had fewer stained cells in the amygdala, mPFC and mPOA than AFR males (Figure 4b). Figure 5 shows a representative stain image from which were made counting.

## Discussion

The results revealed that maternal separation procedure during breastfeeding applied in this study caused significant effects on anxiety-like behaviors and limbic GABA-A receptors immunoreactivity, nevertheless these effect were specific for each gender, data that could be important to understand complex consequences of early stress.

Behaviors were analyzed in three patterns: anxiety, locomotor activity and risk assessment. In relation with first both separated males and females showed an anxiety reduction, but separated females exhibited lesser anxiety behaviors than males: spent more time in open arms (Figure 3a) and head dipping (Figure 3d). Males did not have significant differences in open arm entries, but showed important differences in head dipping; this could indicate that anxiety reduction was not sufficient to let them explore the open arms. The before pattern of anxiety reduction in maternal separated animals was similar to that found by Eklund and Arborelius [14] and Slotten *et al*. [31], who used a similar MS protocol, however, no gender differences were observed in the Eklund and Arborelius study. This may be because they only assessed defensive avoidance, and the male differences in our research lay in other patterns.

Others studies has found opposite results, in which maternal separation stress produce anxiety increase in adult rats [36]–[38], the differences between above studies and our results may be associated with the separation protocol, in studies in which increase anxiety the separation is made for 3 hour daily during two or three weeks while we separated the rats for six hour daily during three weeks. In addition, it should be noted that rat strain used could have contributed to the differences between our data and other studies [3]. Some authors have remarked on the discrepancies in MS studies using Wistar rats, which may be prone to showing an anxiolytic profile [39], [40].

Maternally separated animals, in particular females, have more locomotor activity (Fig. 3b). This tendency could be related to greater impulsivity in the MS subjects. High locomotor activity could also be associated with an anxiolytic pattern in the MS females. Likewise, high impulsivity and hyperactivity levels have been reported by Kwak *et* *al.* [24] using the EPM and others tests. Marín and Planeta [41] reported alterations in MS animals only during adolescence, as this effect became lost during adulthood. The main difference concerning the Marín and Planeta study was that they used a 5-h separation over a 5-day period. However, more measurements must be taken with other tests to prove the validity of these findings because the total entries in the plus maze are not a precise indicator of hyperactivity or impulsivity [42].

Regarding of risk assessment patterns, males exposed to MS visit for more time the center maze and made more stretching behavior than ARF males and all females (Figure 3c and 3d), this result is particularly interesting and has not been reported in previous MS research. The risk assessment has been associated with an conflict between tow unconditioned tendencies; unsafe spaces avoidance and news spaces exploration [15], [43], animals that exhibited more risk assessment behaviors have troubles to make decision, what may be associated with a lower anxiety level that those that stayed more time in close arms [44], but greater than those exploring open arms.

Immunohistochemical analysis indicated that the MS females had lower immunoreactivity for the GABA-A receptor α1 subunit in CA3 region of the hippocampus, prelimbic mPFC, mPOA and hypothalamic pPVN compared to AFR females, while MS males presented lower immunoreactivity in amygdala, mPFC and pPVN. The main GABAergic signal within the hippocampus comes from local interneurons [45], forming part of the local modulation circuits. The reduction in the number of immunostained cells in the CA3 region of the hippocampus could result in an increase in glutamatergic excitatory output to the limbic areas that mediate hypothalamic pPVN inhibition. The greatest concentration of GABA-A receptors in the mPFC are located in layer 3 of the pyramidal cells, which primarily receive inhibitory input from adjacent interneuron layers with addition input from the hippocampus and basal forebrain [46]. GABA-A receptor reduction within the mPFC could result in poor inhibition of the pyramidal neurons followed by a stronger excitatory signal on the limbic nucleus. The reduction in prefrontal GABA-A receptors observed in MS females could contribute to their lower anxiety profile via increased pPVN neuron inhibition resulting in a low HPA response, what coincide with no-published result in our lab, where found a reduction in baseline plasma CORT levels in MS females.

On the other hand, number of immunostained cells reduction in the amygdala could suggest altered GABAergic input from the local interneurons that receive excitatory input, primarily from the PFC and hippocampus [47]. This could be followed by increased GABAergic output and a resulting increase in the inhibition of limbic areas, such as the intermediated nucleus, which, in turn, would result in lower tonic pPVN inhibition. This might explain the making decision conflict observed in MS males, likely related with tow influences: greater excitation by the mPFC and greater inhibition by the amygdala.

In the present work, we observed that the number of immunolabeled cells was significantly reduced in all MS subjects, with the reduction being more evident in males than females. However, the POA was the only structure in which the difference between males and females was statistically significant. The females were more susceptible to receptor subunit loss than males. The POA has large GABAergic activity with a high percentage of neurons synthesizing GABA and expressing GABA-A receptors. This area contains of a dense mesh of local loops receiving and sending GABAergic signals [48]. Most of the inhibitory GABAergic inputs come from the amygdala [17], and a reduction in GABA-A receptors could indicate inadequate local and amygdalar inhibition and a consequent increase in the inhibitory hypothalamic pPVN neuron output. Adding this to the PFC and hippocampus effects could result in strengthening the PVN inhibition and thus explain the low anxiety level shown by the MS females. The gender differences observed in the mPOA could be mediated by sexual hormones. There is evidence that estrogen receptor activation can modify the transcription of different GABA-A subunits [49]. It is interesting to note that maternal behavior, like lordosis and nest building, are affected by local POA GABA transmission [50]. Hence, the MS effects on GABA-A receptors in the POA may be associated with non-genomic transmission of anomalous maternal behavior [51].

Hypothalamic pPVN neurons receive important inhibitory signals from limbic structures and have local inhibitory activity based on essential GABA-A receptors. MS stress is associated with fewer GABA-A receptors in females, which could have reduced the high level of inhibition found in this area; however, further studies are needed to assess whether the GABA input in the pPNV is greater than the GABA-A receptor reduction, resulting in a low HPA axis response pattern.

Other studies have found related MS effects on GABA-A neurotransmission, Hsu *et al*., [52] reported that MS induced changes in hippocampal long-term GABA-A receptor function and subunit expression. Ziabreva *et al*. [53] also reported GABA-A receptor down-regulation in the amygdala and hippocampus in Octodon degus pups separated from their parents. Caldji *et al*. [10] found that neglected maternal care reduced mPFC and amygdala benzodiazepine binding efficiency to GABA-A receptors. Jaworski *et al*. [54] concluded that rats exposed to MS for 15 min showed an increase in GABA-A receptors compared to rats separated for 180 min. Garrett and Wellman [55] found that males were more susceptible than females to mPFC alterations after early chronic stress. The MS effects on GABA transmission could be the associated with the glucocorticoids chronic exposition [19], [56]–[58] and changes in BDNF levels [59] during neurodevelopment.

This study focused on the α1 subunit, however the physiological and behavioral effects observed could be related with changes in others subunits like α2 or α3, α1 subunit has been related with amnesic and sedative benzodiazepine interactions, but α2 and α3 are more strongly associated with anxiolitic performance. It should be noted that the α1 subunit reduction in the MS animals could imply an increase in α2 subunit expression, mainly in the hippocampus [52]. Studies using knock-out mice for the α1 subunit have shown that α1 depletion produces insensitivity to BZ but not to GABA, and its effects were not anxiolytic [60]. Ye *et al*. [61] found that mice with a α1 mutation presented a heightened anxiety profile, but the BZ anxiolytic effect was lost in mice with a α2 mutation but not in α3 mutation [62]. The extended localization of α2 in the limbic system supports its role in anxiety [63]. The changes in different GABA_A_ subunits should thus be studied to gain knowledge about the influence of early experiences on GABA transmission and its effects on anxiety behavior, motor activity and endocrine response.

Separation from dams could have generated chronic stress in the pups, which increase Glu in nervous central system affecting brain development. However, is important take in account the stress hyporesponsive period (SHRP), which occur between 3 and 14 postnatal day, Bock and coworkers [64] showed that MS stress consequences on brain plasticity are dependent of dam separation moment, prior and after to SHRP take place the main effects, thus specifics limbic changes that were exhibit in separated animals, could happened at different neurodevelopment moments. In addition, SHRP may be modified by maternal experiences, to ensure low stress hyporesponsivity is needed maternal care; and separation could induce high stress, even during the SHRP [65], [66]. Results of Dent and coworkers [25] support the hypothesis that the early stress specifics effects on GABA circuit development are exerted mainly out of the SHRP.

In this study, we did not assess the effect of early stress on the function of other neurotransmitter systems that are important for central stress control. Several articles have reported important alterations in serotonin [67], [68], dopamine [69], [36] and adrenaline, which are associated with a wide range of behavioral disruptions. Future studies are needed to differentiate the specific effects of maternal separation on these diverse neurotransmitter systems.

The findings in the present work could be relevant to understanding how early experiences shape behavioral, endocrine and neural profiles, which could be associated with dysfunctions in fitness and mental pathology. This study demonstrated that early influences are not equal in each gender. In fact, patterns found could be related to the epidemiological distribution of mental illness in humans. For example, women usually present more anxiety and mood disorders, while men display more behavioral problems and attention-deficit hyperactive disorders.

## References

[1] Heim C, Nemeroff CB (2001) The role of childhood trauma in the neurobiology of mood and anxiety disorders: preclinical and clinical studies. Biological psychiatry 49: 1023–39. 11430844 10.1016/s0006-3223(01)01157-x

[2] Harlow HF, Dodsworth RO, Harlow MK (1965) Total social isolation in monkeys. Proceedings of the National Academy of Sciences of the United States of America 54: 90–7. 4955132 10.1073/pnas.54.1.90 PMC285801

[3] Levine S (2005) Developmental determinants of sensitivity and resistance to stress. Psychoneuroendocrinology 30: 939–46. 15958281 10.1016/j.psyneuen.2005.03.013

[4] Schable S, Poeggel G, Braun K, Gruss M (2007) Long-term consequences of early experience on adult avoidance learning in female rats: role of the dopaminergic system. Neurobiology of learning and memory 87: 109–22. 16938473 10.1016/j.nlm.2006.07.005

[5] Bock J, Braun K (2011) The impact of perinatal stress on the functional maturation of prefronto-cortical synaptic circuits: Implications for the pathophysiology of ADHD?. Prog Brain 6. Res 189: 155–169. 10.1016/B978-0-444-53884-0.00023-3 21489388

[6] Cicchetti D, Manly JT (2001) Operationalizing child maltreatment: developmental processes and outcomes. Development and psychopathology 13: 755–7. 11771906

[7] Putnam FW (2003) Ten-year research update review: child sexual abuse. Journal of the American Academy of Child and Adolescent Psychiatry 42: 269–78. 12595779 10.1097/00004583-200303000-00006

[8] McCrory E, De Brito SA, Viding E (2010) Research review: The neurobiology and genetics of maltreatment and adversity. J Child Psychol Psychiatry 51: 1079–95. 20546078 10.1111/j.1469-7610.2010.02271.x

[9] Hofer MA (1975) Studies on how early maternal separation produces behavioral change in young rats. Psychosomatic medicine 37: 245–64. 1178795 10.1097/00006842-197505000-00003

[10] Caldji C, Diorio J, Meaney MJ (2000) Variations in maternal care in infancy regulate the development of stress reactivity. Biological psychiatry 48: 1164–74. 11137058 10.1016/s0006-3223(00)01084-2

[11] Caldji C, Francis D, Sharma S, Plotsky PM, Meaney MJ (2000) The effects of early rearing environment on the development of GABAA and central benzodiazepine receptor levels and novelty-induced fearfulness in the rat. Neuropsychopharmacology : official publication of the American College of Neuropsychopharmacology 22: 219–29. 10693149 10.1016/S0893-133X(99)00110-4

[12] Liu D, Caldji C, Sharma S, Plotsky PM, Meaney MJ (2000) Influence of neonatal rearing conditions on stress-induced adrenocorticotropin responses and norepinepherine release in the hypothalamic paraventricular nucleus. Journal of neuroendocrinology 12: 5–12. 10692138 10.1046/j.1365-2826.2000.00422.x

[13] Shalev U, Kafkafi N (2002) Repeated maternal separation does not alter sucrose-reinforced and open-field behaviors. Pharmacology, biochemistry, and behavior 73: 115–22. 10.1016/s0091-3057(02)00756-6 12076730

[14] Eklund MB, Arborelius L (2006) Twice daily long maternal separations in Wistar rats decreases anxiety-like behaviour in females but does not affect males. Behavioural brain research (172) 278–85. 10.1016/j.bbr.2006.05.015 16780968

[15] León R Diego A, Dueñas Zulma J (2012) Efectos de la separación materna temprana sobre el desempeño en el laberinto en cruz elevado en ratas adultas. Acta Biol. Colomb 17: 129–142.

[16] Ulrich-Lai YM, Herman JP (2009) Neural regulation of endocrine and autonomic stress responses. Nature reviews Neuroscience 10: 397–409. 19469025 10.1038/nrn2647 PMC4240627

[17] Jankord R, Herman JP (2008) Limbic regulation of hypothalamo-pituitary-adrenocortical function during acute and chronic stress. Annals of the New York Academy of Sciences 1148: 64–73. 19120092 10.1196/annals.1410.012 PMC2637449

[18] Andersen SL, Teicher MH (2004) Delayed effects of early stress on hippocampal development. Neuropsychopharmacology: official publication of the American College of Neuropsychopharmacology 29: 1988–93. 15316569 10.1038/sj.npp.1300528

[19] Mirescu C, Peters JD, Gould E (2004) Early life experience alters response of adult neurogenesis to stress. Nature neuroscience 7: 841–6. 15273691 10.1038/nn1290

[20] Huot RL, Plotsky PM, Lenox RH, McNamara RK (2002) Neonatal maternal separation reduces hippocampal mossy fiber density in adult Long Evans rats. Brain research 950: 52–63. 12231228 10.1016/s0006-8993(02)02985-2

[21] Renard GM, Rivarola MA, Suarez MM (2010) Gender-dependent effects of early maternal separation and variable chronic stress on vasopressinergic activity and glucocorticoid receptor expr-ession in adult rats. Developmental neuroscience 32: 71–80. 20389078 10.1159/000280102

[22] Faure J, Uys JD, Marais L, Stein DJ, Daniels WM (2006) Early maternal separation followed by later stressors leads to dysregulation of the HPA-axis and increases in hippocampal NGF and NT-3 levels in a rat model. Metabolic brain disease 21: 181–88. 16850259 10.1007/s11011-006-9013-6

[23] Romeo RD, Fossella JA, Bateup HS, Sisti HM, Brake WG, et al (2004) Maternal separation suppresses TGF alpha mRNA expression in the prefrontal cortex of male and female neonatal C57BL/6 mice. Brain research Developmental brain research 152: 73–7. 15283997 10.1016/j.devbrainres.2004.05.007

[24] Kwak HR, Lee JW, Kwon KJ, Kang CD, Cheong IY, et al (2009) Maternal social separation of adolescent rats induces hyperactivity and anxiolytic behavior. The Korean journal of physiology & pharmacology: official journal of the Korean Physiological Society and the Korean Society of Pharmacology 13: 79–83. 10.4196/kjpp.2009.13.2.79 PMC2766697 19885001

[25] Dent G, Choi DC, Herman JP, Levine S (2007) GABAergic circuits and the stress hyporesponsive period in the rat: ontogeny of glutamic acid decarboxylase (GAD) 67 mRNA expression in limbic-hypothalamic stress pathways. Brain research 1138: 1–9. 17276416 10.1016/j.brainres.2006.04.082

[26] Stevenson CW, Halliday DM, Marsden CA, Mason R (2008) Early life programming of hemispheric lateralization and synchronization in the adult medial prefrontal cortex. Neuroscience 155: 852–63. 18634856 10.1016/j.neuroscience.2008.06.013

[27] Sanchez MM, Aguado F, Sanchez-Toscano F, Saphier D (1995) Effects of prolonged social isolation on responses of neurons in the bed nucleus of the stria terminalis, preoptic area, and hypothalamic paraventricular nucleus to stimulation of the medial amygdala. Psychoneuroendocrinology 20: 525–41. 7675937 10.1016/0306-4530(94)00083-m

[28] Camozzato TS, Winkelmann-Duarte EC, Padilha CB, Miguel SP, Bonzanini L, et al (2009) Neonatal handling reduces the number of cells in the medial preoptic area of female rats. Brain research 1247: 92–9. 18977206 10.1016/j.brainres.2008.09.077

[29] Lephart ED, Watson MA (1999) Maternal separation: hypothalamic-preoptic area and hippocampal calbindin-D28K and calretinin in male and female infantile rats. Neuroscience letters 267: 41–4. 10400244 10.1016/s0304-3940(99)00326-2

[30] Farkas J, Reglodi D, Gaszner B, Szogyi D, Horvath G, et al (2009) Brain Res Bull 29. 79(3–4): 208–14. 10.1016/j.brainresbull.2008.12.011 19150489

[31] Slotten HA, Kalinichev M, Hagan JJ, Marsden CA, Fone KC (2006) Long-lasting changes in behavioural and neuroendocrine indices in the rat following neonatal maternal separation: gender-dependent effects. Brain research (1097 123–32. 10.1016/j.brainres.2006.04.066 16730678

[32] Hofer MA (1994) Early relationships as regulators of infant physiology and behavior. Acta Paediatrics Supplement. 397: 9. 10.1111/j.1651-2227.1994.tb13260.x 7981480

[33] Albrechet-Souza L, Borelli KG, Brandao ML (2008) Activity of the medial prefrontal cortex and amygdala underlies one-trial tolerance of rats in the elevated plus-maze. Journal of neuroscience methods. 169: 109–18. 10.1016/j.jneumeth.2007.11.025 18190969

[34] Rodgers RJ, Haller J, Holmes A, Halasz J, Walton TJ, et al (1999) Corticosterone response to the plus-maze: high correlation with risk assessment in rats and mice. Physiology & behavior 68: 47–53. 10627061 10.1016/s0031-9384(99)00140-7

[35] Paxinos (2004) The Rat Nervous System. San Diego-California: Elsevier Academic Press.

[36] Aisa B, Tordera R, Lasheras B, Del Río J, Ramírez MJ (2007) Cognitive impairment associated to HPA axis hyperactivity after maternal separation in rats. Psychoneuroendocrinology, 32(3), 256–266. 10.1016/j.psyneuen.2006.12.013 17307298

[37] Daniels WM, Pietersen CY, Carstens ME, Stein DJ (2004) Maternal separation in rats leads to anxiety-like behavior and a blunted ACTH response and altered neurotransmitter levels in response to a subsequent stressor. Met Brain Dis. 19: 3–14. 10.1023/b:mebr.0000027412.19664.b3 15214501

[38] Mcintosh J, Anisman H, Merali Z (1999) Short- and long-periods of neonatal maternal separation differentially affect anxiety and feeding in adult rats, gender dependent effects. Brain Res Dev Brain Res. 113: 97–106. 10.1016/s0165-3806(99)00005-x 10064879

[39] Ploj K, Roman E, Nylander I (2002) Effects of maternal separation on brain nociceptin/orphanin FQ peptide levels in male Wistar rats. Pharmacology, biochemistry, and behavior 73: 123–9. 10.1016/s0091-3057(02)00778-5 12076731

[40] Roman E, Gustafsson L, Berg M, Nylander I (2006) Behavioral profiles and stress-induced corticosteroid secretion in male Wistar rats subjected to short and prolonged periods of maternal separation. Hormones and behavior 50: 736–47. 16876800 10.1016/j.yhbeh.2006.06.016

[41] Marin MT, Planeta CS (2004) Maternal separation affects cocaine-induced locomotion and response to novelty in adolescent, but not in adult rats. Brain research. 1013: 83–90. 10.1016/j.brainres.2004.04.003 15196970

[42] Dawson GR, Crawford SP, Stanhope KJ, Iversen SD, Tricklebank MD (1994) One-trial tolerance to the effects of chlordiazepoxide on the elevated plus maze may be due to locomotor habituation, not repeated drug exposure. Psychopharmacology. 113: 570–2. 10.1007/BF02245242 7862878

[43] Rogers RJ, Johnson JT (1995) Factor analysis of spaciotemporal and ethological measures in the murine elevated plus-maze test of anxiety. Pharmacology biochemistry and behavior 52 (2): 297–303. 10.1016/0091-3057(95)00138-m 8577794

[44] Blanchard DC, Griebel G, Blanchard RJ (2001) Mouse defensive behaviors: Pharmacological and behavioral assays for anxiety and panic. Neuroscience and Biobehavioral Reviews 25: 205–218. 11378177 10.1016/s0149-7634(01)00009-4

[45] Banke TG, McBain CJ (2006) GABAergic input onto CA3 hippocampal interneurons remains shunting throughout development. The Journal of neuroscience: the official journal of the Society for Neuroscience 26: 11720–5. 17093093 10.1523/JNEUROSCI.2887-06.2006 PMC6674795

[46] Hashimoto T, Arion D, Unger T, Maldonado-Aviles JG, Morris HM, et al (2008) Alterations in GABA-related transcriptome in the dorsolateral prefrontal cortex of subjects with schizophrenia. Molecular psychiatry 13: 147–61. 17471287 10.1038/sj.mp.4002011 PMC2882638

[47] Sah P, Faber ES, Lopez De Armentia M, Power J (2003) The amygdaloid complex: anatomy and physiology. Physiological reviews 83: 803–34. 12843409 10.1152/physrev.00002.2003

[48] Uchida S, Noda E, Kakazu Y, Mizoguchi Y, Akaike N, et al (2002) Allopregnanolone enhancement of GABAergic transmission in rat medial preoptic area neurons. American journal of physiology Endocrinology and metabolism 283: E1257–65. 12424107 10.1152/ajpendo.00049.2002

[49] Herbison AE, Augood SJ, Simonian SX, Chapman C (1995) Regulation of GABA transporter activity and mRNA expression by estrogen in rat preoptic area. The Journal of neuroscience : the official journal of the Society for Neuroscience 15: 8302–9. 8613763 10.1523/JNEUROSCI.15-12-08302.1995 PMC6577950

[50] Arrati PG, Carmona C, Dominguez G, Beyer C, Rosenblatt JS (2006) GABA receptor agonists in the medial preoptic area and maternal behavior in lactating rats. Physiology & behavior 87: 51–65. 16297940 10.1016/j.physbeh.2005.08.048

[51] Francis D, Diorio J, Liu D, Meaney MJ (1999) Nongenomic transmission across generations of maternal behavior and stress responses in the rat. Science 286: 1155–8. 10550053 10.1126/science.286.5442.1155

[52] Hsu FC, Zhang GJ, Raol YS, Valentino RJ, Coulter DA, et al (2003) Repeated neonatal handling with maternal separation permanently alters hippocampal GABAA receptors and behavioral stress responses. Proceedings of the National Academy of Sciences of the United States of America 100: 12213–8. 14530409 10.1073/pnas.2131679100 PMC218738

[53] Ziabreva I, Poeggel G, Schnabel R, Braun K (2003) Separation-induced receptor changes in the hippocampus and amygdala of Octodon degus: influence of maternal vocalizations. The Journal of neuroscience : the official journal of the Society for Neuroscience 23: 5329–36. 12832558 10.1523/JNEUROSCI.23-12-05329.2003 PMC6741186

[54] Jaworski JN, Francis DD, Brommer CL, Morgan ET, Kuhar MJ (2005) Effects of early maternal separation on ethanol intake, GABA receptors and metabolizing enzymes in adult rats. Psychopharmacology 181: 8–15. 15830234 10.1007/s00213-005-2232-4

[55] Garrett JE, Wellman CL (2009) Chronic stress effects on dendritic morphology in medial prefrontal cortex: sex differences and estrogen dependence. Neuroscience 162: 195–207. 19401219 10.1016/j.neuroscience.2009.04.057 PMC2720075

[56] Bredy TW, Grant RJ, Champagne DL, Meaney MJ (2003) Maternal care influences neuronal survival in the hippocampus of the rat. The European journal of neuroscience 18: 2903–9. 14656341 10.1111/j.1460-9568.2003.02965.x

[57] Chung EK, Bian ZX, Xu HX, Sung JJ (2009) Neonatal maternal separation increases brain-derived neurotrophic factor and tyrosine kinase receptor B expression in the descending pain modulatory system. Neuro-Signals. 2009 17: 213–21. 10.1159/000224631 19546592

[58] Pham K, Nacher J, Hof PR, McEwen BS (2003) Repeated restraint stress suppresses neurogenesis and induces biphasic PSA-NCAM expression in the adult rat dentate gyrus. The European journal of neuroscience 17: 879–86. 12603278 10.1046/j.1460-9568.2003.02513.x

[59] Lippmann M, Bress A, Nemeroff CB, Plotsky PM, Monteggia LM (2007) Long-term behavioural and molecular alterations associated with maternal separation in rats. The European journal of neuroscience 25: 3091–8. 17561822 10.1111/j.1460-9568.2007.05522.x

[60] Tobler I, Kopp C, Deboer T, Rudolph U (2001) Diazepam-induced changes in sleep: role of the alpha 1 GABA(A) receptor subtype. Proceedings of the National Academy of Sciences of the United States of America 98: 6464–9. 11353839 10.1073/pnas.111055398 PMC33491

[61] Ye G, Baker K, Mason SM, Zhang W, Kirkpatrick L, et al (2010) GABAA Receptor α1 Subunit (Gabra1) Knockout Mice: Review and New Results. Neuromethods 44: 65–90.

[62] Low K, Crestani F, Keist R, Benke D, Brunig I, Benson JA (2000) Molecular and neuronal substrate for the selective attenuation of anxiety. Science 290: 131–4. 11021797 10.1126/science.290.5489.131

[63] Nutt DJ, Malizia AL (2001) New insights into the role of the GABA(A)-benzodiazepine receptor in psychiatric disorder. Br J Psychiatry 179: 390–6. 11689393 10.1192/bjp.179.5.390

[64] Bock J, Gruss M, Becker S, Braun K (2005) Experience-induced changes of dendritic spine densities in the prefrontal and sensory cortex: correlation with developmental time windows. Cereb Cortex 15: 802–808. 15371297 10.1093/cercor/bhh181

[65] Faturi CB, Tiba PA, Kawakami SE, Catallani B, Kerstens M, et al (2010) Disruptions of the mother-infant relationship and stress-related behaviours: altered corticosterone secretion does not explain everything. Neuroscience and biobehavioral reviews 34: 821–34. 19751762 10.1016/j.neubiorev.2009.09.002

[66] Lupien SJ, McEwen BS, Gunnar MR, Heim C (2009) Effects of stress throughout the lifespan on the brain, behaviour and cognition. Nat Rev Neurosci 10: 434–445. 19401723 10.1038/nrn2639

[67] Arborelius L, Eklund MB (2007) Both long and brief maternal separation produces persistent changes in tissue levels of brain monoamines in middle-aged female rats. Neuroscience 145: 738–50. 17222517 10.1016/j.neuroscience.2006.12.007

[68] Leventopoulos M, Russig H, Feldon J, Pryce CR, Opacka-Juffry J (2009) Early deprivation leads to long-term reductions in motivation for reward and 5-HT1A binding and both effects are reversed by fluoxetine. Neuropharmacology 56: 692–701. 19138691 10.1016/j.neuropharm.2008.12.005

[69] Huot RL, Thrivikraman KV, Meaney MJ, Plotsky PM (2001) Development of adult ethanol preference and anxiety as a consequence of neonatal maternal separation in Long Evans rats and reversal with antidepressant treatment. Psychopharmacology 158: 366–73. 11797057 10.1007/s002130100701

